# The Social Determinants of Health: Time to Re-Think?

**DOI:** 10.3390/ijerph17165856

**Published:** 2020-08-12

**Authors:** John Frank, Thomas Abel, Stefano Campostrini, Sarah Cook, Vivian K. Lin, David V. McQueen

**Affiliations:** 1 Director of Knowledge Exchange and Research Impact, Usher Institute, University of Edinburgh, Room 1-308, Doorway #1, Teviot Hall, Teviot Place, Edinburgh EH8 9AG, UK; 2 ISPM, University of Bern, 3012 Bern, Switzerland; thomas.abel@ispm.unibe.ch (T.A.); dvmcqueengc@comcast.net (D.V.M.); 3 Department of Economics, University Ca’ Foscari Venice, 30121 Venice, Italy; stefano.campostrini@unive.it; 4 Director, Institute for Global Development, University of New South Wales, John Goodsell Building, Sydney, NSW 2052, Australia; s.cook@unsw.edu.au; 5 Executive Associate Dean, Faculty of Medicine, University of Hong Kong, Hong Kong, China; vklin@hku.hk

**Keywords:** social determinants of health, health promotion, health policy

## Abstract

Twelve years have now passed since the influential WHO Report on the Social Determinants of Health (SDoH) in 2008. A group of senior international public health scholars and decision-makers met in Italy in mid-2019 to review the legacy of the SDoH conceptual framework and its adequacy for the many challenges facing our field as we enter the 2020s. Four major categories of challenges were identified: emerging “exogenous” challenges to global health equity, challenges related to weak policy and practice implementation, more fundamental challenges related to SDoH theory and research, and broader issues around modern research in general. Each of these categories is discussed, and potential solutions offered. We conclude that although the SDoH framework is still a worthy core platform for public health research, policy, and practice, the time is ripe for significant evolution.

## 1. Purpose/Rationale

The underlying concept of the social determinants of health (SDoH) has a long public health history [1]. More recently, the WHO Commission on SDoH Report of 2008 [2,3] represented a major milestone in that history, reaching a much wider public than previously. Since this landmark report, much research has attempted to document the impact of SDoH thinking on health policy, health disparities, and public health practice. However, many prominent observers have judged “the health gap” across social classes not to be closing in most societies over the last decade [4,5,6,7]. Strikingly, despite this negative finding, few researchers—McQueen, Crammond and Carey, and Schrecker being notable exceptions [8,9,10]—appear to have seriously questioned the conceptual SDoH framework itself.

The international authors of this paper held a think tank in the summer of 2019 to discuss potential limitations of the traditional SDoH framework and possible approaches to updating it. This paper summarizes their thoughts, emphasizing areas of agreement. The aim of the group was not to critique SDoH concepts purely for the sake of being critical (though we appreciate that many academics would find that laudable). Nor did we reach the point of proposing a new approach to “replace” the SDoH framework, which has been a helpful beacon for progressive public health policy and practice for a dozen years. Rather, in this paper we identify some emerging global issues as well as theoretical and methodological critiques of the SDoH conceptual framework that, in our view, should provoke public health professionals and researchers to rethink that framework. In sum, we identify 15 emerging global issues across four broad categories that provoke us to ask whether the SDoH framework ought to be rethought and updated. We believe this is just the beginning of a long and complex process necessarily involving many diverse international stakeholders, for which we only hope to provide the stimulus.

## 2. Previous Critical Commentaries on the SDoH Approach

In his 2009 editorial [8] “Three challenges for the social determinants of health pursuit,” McQueen identifies three specific challenges facing those who would implement the SDoH perspective:

1. The foundational basis for the SDoH approach is “ancient,” with written materials dating back hundreds of years, documenting the ubiquity of health inequalities by socioeconomic status. This in turn means that it is very difficult to “create a sense of urgency” to address these perennial phenomena observed in virtually all human societies. Indeed, the biblical observation that “the poor are always with us” (Deuteronomy 15) captures the kind of inured and complacent audience reaction which frequently occurs when SDoH ideas are presented, especially to policy stakeholders.
2. The measurement of “what is social” is problematic. McQueen points to a persistent lack of convincing conceptual work in public health publications around the complex construct of social class and its profound nonequivalence to simple unidimensional markers such as individual income or education, let alone area-based measures such as indices of multiple deprivation, which can have remarkably different implications in different settings.
3. The etiological evidence base is relatively well-developed for social determinants as key *causal factors* affecting health status. However, the evidence base for the effectiveness of specific public health *interventions* (programs and policies) to reduce overall ill health in a society, while also reducing health inequalities by socioeconomic status (SES), is much less well developed [11].

Crammond and Carey, writing in 2017 [9] for a social-science audience, point out that policy-making to reduce health inequalities is essentially a normative process, requiring more explicitly political analysis and activity rather than merely empirically based efforts to assemble “evidence” for decision-making. Yet, most research to support SDoH-based policies and programmes has been overwhelmingly framed as “evidence” [2,3,4,5].

Schrecker [10], writing a decade after McQueen, calls attention to the “hard (difficult) politics of inequality,” which he blames for much of the apparent ineffectiveness of policies in several countries which have at least tried to reduce socioeconomic inequalities and related health disparities. He is particularly convincing on the role of international trade agreements in maintaining both economic and health inequalities, combined with transnational corporations’ normal operating methods—including shifting profits/jobs to lower-tax/pay settings, aided and abetted by the shadowy world of global tax shelters. Such forces have been conspiring for decades to make it harder and harder for even the most committed national governments to actually reduce socioeconomic inequalities “in their own backyards”—indeed, the fences around those backyards are now completely porous. We pick this topic up again below in identifying missing elements in traditional (pre-2009) SDoH writings that are important considerations today in public health policy-making and practice.

## 3. Emerging Challenges to the SDoH Model

We see the following major challenges to traditional SDoH thinking that have either grown or emerged over time:

### 3.1. Emerging “Exogenous” Challenges to Global Health Equity

1. *Emergence and recognition of ever more complex and “wicked” public health problems.* For example, the obesity pandemic continues unabated in high-income countries (HICs) and is rapidly growing in lower- and middle-income settings (LMICs), despite widespread policy and program efforts to control it. One challenge to SDoH thinking is that obesity is not consistently socially patterned internationally. Indeed, obesity tends to demonstrate “reverse social gradients” in some HIC adult male populations, and more widely in LMICs [12]. This suggests that SDoH concepts are not well-suited to helping public health professionals and policy makers deal effectively with obesity at a global scale. As the widely cited Foresight Report on Obesity in the UK pointed out more than a decade ago [13], using the word “determinants” in relation to such a complex, system-level problem seems problematically reductionistic.
2. *In a similar vein, over the last decade there has been increasing recognition of the importance of government legislation/regulation to control “marketable health hazards”* to combat the ongoing global rise in noncommunicable/chronic diseases. Such hazards include poor-quality and energy-dense food and drink, alcohol, tobacco and psychoactive drugs, and gambling (especially online) [14,15,16]. Yet, typical SDoH writings appear unclear—or at least uninformed—about the specific sections and levels of government at which such decisions are actually taken, making targeted, effective advocacy more difficult.
3. *Increased multisectorality of global health and development thinking:* The UN Sustainable Development Goals (SDGs) cover many sectors. On the one hand, health is explicitly prominent in only 1 of its 17 Goals. On the other hand, in line with the “health in all policies” terminology, health is typically interpreted by public health advocates as inherent in all of the SDGs. This is an expansion of those SDoH writings that can be read as assuming that all societies should value health outcomes above others—so-called “health-ism.” This issue is extensively discussed by Crammond and Carey [9].
4. *Increasing scientific evidence and public recognition that socioeconomic inequality, per se, probably has a causal relationship to the suboptimal “performance” of many societies globally,* extending well beyond health outcomes [17,18,19,20,21]. This recognition has led to an increased emphasis on equity-oriented public policy-making, with a focus on redistributive tax and transfer (welfare benefits) policies as key levers for achieving increased societal inequity [22,23,24]. SDoH writings before 2009 often do not prioritize tax and transfer policies in this way.
5. *Increased recognition by the global public health community that climate change and environmental degradation (barely mentioned in the main SDoH writings) are core priorities for public health,* in partnership with many others in civil society. Fortunately, it is quite possible to update SDoH thinking to incorporate the repeated observation that the poorest and least educated persons in any society are most affected by incipient climate change and environmental degradation, so that, in fact, the environmental justice movement fits well into SDoH concepts [25].
6. *SDoH approaches tend to avoid the “relationality” in the unequal distribution of resources for health.* SDoH thinking tends to focus on “deprivation,” saying nothing about the privileged side. However, insofar as health inequalities are socially made or reproduced (i.e., they are preventable “inequities”), their generation and perpetuation represent an issue of political power and economic influence [19,20,21]. SDoH writings traditionally appear to avoid confronting this issue head-on, perhaps for strategic reasons related to the conservative political environment during the years after the WHO Commission Report in 2008, which, of course, coincided with the 2008 recession.

### 3.2. Challenges Related to Weak Policy and Practice Implementation

There are recurring lacunae among publications expounding the traditional SDoH model concerning the most frequent “pitfalls” and threats to the actual implementation of effective and widely acceptable policies and programs to improve health equitably in the real world:

1. *Lack of in-depth analysis of institutions’ key role in codetermining how well-intended, pro-equity policies and programs are finally executed in communities:* Institutions—whether NGOs, government-based, or academic—all have a role in supporting, both in spirit and financially, key areas of SDoH work. For example, the WHO played a key role in creating excitement about the SDoH with the Marmot report (2005–8). However, the WHO, like all large global institutions, is a microcosm of diverse ministries of health and political leaders of all types who constantly want to move on to new agendas. It thus sometimes appears to act rapidly onto the next exciting idea before the last one has been fully implemented. Such influential institutional behaviours are worthy of more study.
2. *Institutions are often affected by the overarching influence of cultural and political beliefs and related national politics:* In the USA’s Healthy People 2020 planning process, for example, the SDoH played a role, but it was minor. Even before the Trump period, there was a distinct lack of enthusiasm for SDoH ideas at the top of US public health leadership, a situation that could only get worse under the current government. Notably, there has been a sharp falloff of support for SDoH in the USA, reflected in rising right-wing political activities globally, which signals a move away from the “social.” Lack of financial support for SDoH policies then follows in lockstep [26].
3. *Insufficient attention to the precise public sector context for intervention (i.e., the precise sections and levels of government most likely to be able to action policy and program advice in a given setting concerning SDoH):* As mentioned in the previous section, providing advice to public health professionals on interventions to improve health equitably without clearly specifying which section and level of government should be the priority target of advocacy efforts can seriously impair those advocates’ effectiveness. For example, the radical 2012 Lansley reforms of England’s previously NHS-based public health system were sold by the Tory-led coalition government on the basis that there would be clear population health benefits from relocating public health services out of the NHS to some 200 local authorities (LAs) across England. Specifically, it was argued that this reorganisation would enable those professionals to have more access to decisions made by local authorities (LAs), such as zoning and licensing of fast-food or alcohol/tobacco outlets, potentially having a strong impact on NCD risk factors in an equitable way. That argument systematically avoided pointing out that public health professionals immersed in the local bureaucracies of LAs might well be unable to engage simultaneously in effective national-level advocacy. That failure of dual tasking by public health might in turn be expected to reduce the chances of successful control of noncommunicable disease risk factors through national-level legislation or regulation, as has been advocated by many experts [14,15,16]. This is particularly the case for increased taxes on unhealthy foods and drinks or subsidies on healthy ones—policy options now widely advocated by public health experts [15], which necessarily require advocacy at the national level aimed at that level of government, which largely controls such taxes and transfers—certainly within the UK.
4. *Innovative approaches to the governance of programs/policies/interventions:* Given the deep complexity of many current public health challenges (e.g., the obesity pandemic) and the need to involve multiple actors of “pluralistic society,” it is essential to innovate public/voluntary sector governance in order to avoid “pouring new wine into old wineskins.” The cocreation approach [27,28,29], for instance, accepts this complexity and the several “multis” linked to it (multilevel, multidisciplinary, multi-actors, etc.). This approach focuses on searching for common objectives across the typically diverse stakeholder constituencies with an interest in such challenges.
5. *Weak analysis of, and planning for, powerful and sophisticated political opposition to proequity policies and programs:* In many pre-2009 writings about SDoH, there appears to be a slightly naïve unwillingness to acknowledge the virtual certainty of explicit and profound opposition by powerful vested interests to policies likely to improve health equitably. Policy-analytic approaches originating in political science, including the careful identification of “interest groups” around any proposed legislation or regulation, would better enable public health professionals to plan for, and succeed in overcoming, such daunting opposition. A particularly disturbing example is the protracted resistance on the part of the British food production, processing, marketing, and distribution firms to shift their obesogenic practices that massively contribute to that slow global pandemic [14,15].

### 3.3. More Fundamental Challenges Related to SDoH Theory and Research

There are weaknesses in much public health research, including that intended to support SDoH approaches, which have held back the generation of robust and practical evidence needed to guide policy-making to improve health equitably. These include the following:

1. *SDoH research tends to focus on individuals/populations as more or less passive “victims of deprivation/inequality” and thus “carriers of risks”:* This reflects a limited and rather paternalistic view; more importantly, it leaves unexplored the potential agency of people to use social resources for health. The early SDoH approach included the broader sense of agency by referring to A. Sen’s “capability” approach [30] but later dropped it.
2. *Persistent focus on, and repetition of, mere descriptions of health and other inequalities:* Although some public health research has been brilliant at analysing the underlying origins and drivers of social and economic inequalities in health, the vast bulk of such research rarely goes beyond increasingly more “precise” description. This continued focus on description has led to a paucity of studies on “how and why” to change things—developing and testing feasible policy and program interventions to guide policy makers in improving health equitably. More recently, critical social scientists have provided strong theoretical frameworks to support the development of a new field of “public health intervention research” to address previous shortcomings in this arena [31,32].
3. *Weak research theory and methods for assessing the socioeconomic redistributive effects of public health interventions (i.e., whether they increase or decrease health inequalities):* As an example, a recent review of the degree to which the existing evaluations of all sorts of public health interventions have adhered to widely accepted methodological criteria for assessing an intervention’s differential effects, in this case across different socioeconomic groups, shows that remarkably few studies to date have passed this basic test of quality [9]. Analogously, there has been inadequate development of theory to support public health interventions’ evaluations, especially related to their distributional effects [33]. This weakness is accompanied by widespread failure among public health researchers to understand and address the complex interplay of structural conditions and individual agency [34].
4. *Excessive reliance on traditional scientific ideas about “generalizability” (external validity) of evidence:* Biomedical science, viewing all human beings as members of one “genetically bottlenecked” species, has traditionally tended to assume biological equivalence across human societies for biomedical interventions. Notably, even that is increasingly coming into question—for example, due to humans’ surprising genetic and epigenetic diversity. However, policy and program interventions aimed at changing human behaviour for health gains are typically so contextualized by the sociocultural and political setting of their implementation that their effects (both desired and undesired) may be virtually impossible to extrapolate generally. Fortunately, recent social science innovations in both “realist evaluation” and “complex intervention” methodologies are shifting public health practice towards a more explicit characterization of the *context* of any such interventions, which are the mainstay of chronic disease prevention through behavioural change [35,36]. Explicitly addressing instead of ignoring or controlling context effects could be achieved by more use of parallel case studies of particular settings’ influence on such variation in a given intervention’s effects.
5. *Excessive reliance on randomization in intervention trials:* The randomised controlled trial (RCT) still reigns supreme in the eyes of many public health experts, even when randomization is not feasible or ethical, or more cost-effective, compared with sophisticated quasi-experimental/observational study designs from sociology and economics, which are especially well suited to evaluating policy and program effectiveness across societies and time periods [37,38,39]. However, epidemiology, “the basic science of public health,” has been slow to adopt these new study designs.

### 3.4. Broader Issues around Modern Research

A more fundamental development is that the state of the art in scientific research at large is currently being challenged, and sometimes undermined, by current societal trends. Two such trends are illustrative: research funding and publishing. Research funding bodies—both governmental and private—privilege conservative traditional research, often falling behind in the adoption of newer (but sometimes riskier) research methods. Publishing houses for peer-reviewed research tend to reinforce this traditional conservatism, a point reinforced in our think tank group, which included much editorial experience. Further noted was the now almost complete global dominance of the English language among research funders and publishers, which can perpetuate a conceptually limited range of research models in many fields. The United States, the largest funder of global health research, continues to privilege biomedically oriented investigations. In the public health field, this has contributed to a lack of attention to and interest in complex “messy” questions requiring a more philosophical and historical approach. For example, issues of social and cultural context and the influence on health of different political and governance systems are not given the research attention needed. Multidisciplinary research has become widely acknowledged as important, but it is still not routinely practised because research, funding, and publishing institutions are still more comfortable operating within traditional disciplinary borders.

A current example is the social patterning of COVID-19 cases, hospitalizations, and deaths. Prominent publications have called for more explicit and effective policy consideration of the widely confirmed preponderance of this new burden of illness among the socially disadvantaged, including visible minorities [40]. Yet no policies or programs have been launched in either the UK or the USA to effectively tackle this prominent and profoundly inequitable aspect of the pandemic. It is just as if the relevant research documenting this problem had never been done.

## 4. Conclusions

Without question, the SDoH conceptual framework has positively altered public health thinking since 2008, and it is still very salient to policy and practice. However, its “fit” to current global challenges across sectors requires recalibration—a “rethink.” Many researchers have shown how health inequalities, despite many well-intended policy interventions to reduce them, are increasing almost everywhere. However, very few public health experts have questioned the SDoH theoretical approach itself, which has inspired most such interventions. Here, we have made the first systematic attempt to show the multiple reasons why the SDoH approach now should be revised. We urge national and international public health organizations, as well as the corresponding research, policy, and practice communities, to face this challenge. One recalls the quotation “All models are wrong, some are useful” [41]. No conceptual framework is suitable for indefinite use: times have changed, new challenges have emerged, public health knowledge has improved, and the arsenals of available methods have grown. It is time for changes to the way we think about SDoH. This is particularly urgent in the (post) COVID-19 times, when, both for differential access to care and for the effects of the economic crisis linked to the pandemic, inequalities in health are likely to increase, showing fragilities unthinkable at the time in which the SDoH paradigm was proposed [42].
